# Cross-cultural adaptation and psychometric evaluation of the Portuguese version of the family resilience questionnaire – short form (FaRE-SF-P) in women with breast cancer

**DOI:** 10.3389/fpsyg.2022.1022399

**Published:** 2023-01-17

**Authors:** Sílvia Almeida, Daniel Rodrigues da Silva, Diana Frasquilho, Beatriz Costa, Berta Sousa, Telmo Mourinho Baptista, Jaime Grácio, Raquel Lemos, Albino J. Oliveira-Maia

**Affiliations:** ^1^Champalimaud Research and Clinical Centre, Champalimaud Foundation, Lisbon, Portugal; ^2^Graduate Programme in Clinical and Health Psychology, Faculdade de Psicologia da Universidade de Lisboa, Lisbon, Portugal; ^3^Breast Unit, Champalimaud Clinical Centre, Champalimaud Foundation, Lisbon, Portugal; ^4^Faculdade de Psicologia, Universidade de Lisboa, Lisbon, Portugal; ^5^NOVA Medical School, Faculdade de Ciências Médicas, NMS, FCM, Universidade NOVA de Lisboa, Lisbon, Portugal; ^6^ISPA-Instituto Universitário de Ciências Psicológicas, Sociais e da Vida, Lisbon, Portugal

**Keywords:** family resilience, breast cancer, psychological distress, assessment, coping, validation

## Abstract

**Background:**

A diagnosis of cancer, and the resulting treatment process, can be perceived as a life-threatening event, affecting not only patients but also their social network and, more specifically, their relatives. While the ability to cope and adjust to difficult health situations may be challenging, family resilience may optimize a positive adaptation to adversity and contribute to enhance the patient’s quality of life. The Family Resilience Questionnaire (FaRE) is a self-report measure of family resilience that assesses this construct systematically. We aimed to validate the Portuguese version of a short form of the FaRE (FaRE-SF-P) in a sample of women with breast cancer.

**Methods:**

147 women recently diagnosed with early breast cancer were recruited at the Champalimaud Clinical Centre in Lisbon. Participants completed psychometric assessment including the Portuguese version of the FaRE-SF-P, composed by two subscales of the original version – the FaRE Perceived Family Coping (FaRE-PFC) and the FaRE Communication and Cohesion (FaRE-CC). Confirmatory factor analysis (CFA) was performed to assess the factor structure of the FaRE-SF-P. Construct validity was assessed using the Hospital Anxiety and Depression Scale (HADS) for divergent validity, and the Modified Medical Outcomes Study Social Support Survey (mMOS-SS) as well as the social functioning subscale from the European Organization for Research and Treatment of Cancer Quality of Life Questionnaire-Core 30 (EORTC QLQ-C30) for convergent validity.

**Results:**

The CFA results confirmed a correlated two-factor structure model consistent with the Perceived Family Coping and the Communication and Cohesion subscales. Internal consistency reliability indicated good values both for Perceived Family Coping and Communication and Cohesion subscales. The results for construct validity showed acceptable convergent and divergent validity.

**Discussion:**

The FaRE-SF-P showed good psychometric properties demonstrating to be a valid and reliable family resilience measure to use in Portuguese women diagnosed with breast cancer. Since FaRE-SF-P is a short instrument it may be a useful screening tool in an oncological clinical practice routine.

## Introduction

Resilience is a multidimensional construct, which can be defined as one’s ability to mobilize coping resources to adapt and properly function after a perceived significant adverse event (Southwick et al., 2014). From the several dimensions that may compose the construct of resilience, family resilience is of considerable relevance and can be defined as the ability of a functional system to withstand and adapt to adversity (Walsh, 2021). The importance of this dimension stems from the notion that perceived adverse events occurring to one member impact the whole family, and, in turn, that the dynamic interpersonal processes within the family mediate possible adaptation for the individual members, their relationships, and, finally, the whole system (Walsh, 2021).

Cancer diagnosis and the respective treatment process are normally perceived as life-threatening events (Seiler and Jenewein, 2019), leading to significant levels of distress and, possibly even the development of major depression and other neuropsychiatric disorder (Mitchell et al., 2011; Smith, 2015). Such events affect not only patients but also impact their social network and, more specifically, their relatives (Edwards and Clarke, 2004). In the presence of a life-threatening event such as a cancer diagnosis, both patients and their relatives will need to adapt not only individually but also in terms of their family dynamics (Faccio et al., 2018). Cancer diagnosis and treatment impose to the whole family, as a unit, the need to face new challenges in the different stages of the disease. These challenges can range from adaptation to managing resources between work and added home responsibilities, to changing family roles, or even the need to balance and adapt to the needs of the whole family (Northouse, 1992). Relatives of patients with cancer are also at high risk of developing affective symptoms, with prevalence rates of anxiety and depression in this population ranging from 20 to 40% (Friðriksdóttir et al., 2011).

In that sense, it becomes fundamental to understand which psychosocial factors may play a role in the prevention of these conditions and its burdensome consequences. Resilience, perceived social support (Zhao et al., 2020; Tamura, 2021), perceived family support (Su et al., 2017) and family communication skills (Park et al., 2022) have been reported in the literature as important protective factors for symptoms of depression and for the adaptation to this life-threatening event. To optimize the prevention of these neuropsychiatric disorders, clinicians and researchers need to be attentive, screen and monitor affective symptoms in patients with cancer (Walker et al., 2014) and, additionally, understand and bolster the psychosocial resources available both for patients and their relatives (Seiler and Jenewein, 2019).

For resilience and, specifically, family resilience, Walsh’s conceptual framework has been used in the oncological setting, providing insights on the relevance of such construct for patients and their relatives (Walsh, 2021). Walsh’s *Family Resilience Framework* provides multilevel systems orientation associated with a positive adaptation in the presence of perceived adverse events. Under this framework, three key processes are proposed to underlie functional adaptation of the whole family system to the perceived adversity. First, the family’s belief system, composed of making meaning of adversity, a positive outlook and transcendence and spirituality (Walsh, 2016). The second key process concerns the familial organizational processes, comprised of flexibility, connectedness, and resource mobilization between family members (Walsh, 2016). Finally, the third key process is communication and problem-solving skills, where clarity, open emotional sharing and a collaborative problem-solving approach should be present to optimize a positive adaptation to adversity (Walsh, 2016).

Albeit being a useful theoretical model to guide the understanding of family resilience to adversity, a quantification of this construct is needed, and to do so, adequate psychometric instruments are required. Four psychometric instruments have been developed and submitted to a formal validation process for this purpose, in diverse contexts. The *Family Resilience Assessment Scale* (*FRAS*; Sixbey, 2005) was implemented in the general American population without considering the presence of a significant perceived adverse event, leading to a significant limitation in the process of its validation. The second example is the *Family Resilience Assessment (FRA)*, with its authors suggesting that, throughout the validation process, the items were not all fitting the construct which they were trying to measure, leading to important constraints in the assessment of the family resilience construct (Duncan Lane et al., 2017). The third psychometric instrument, *The Walsh Family Resilience Questionnaire* (*WFRQ*; Walsh, 2016) was designed by the developer of the family resilience construct framework considered above. However, validation of this psychometric instrument was not conducted in an oncological setting, with one study conducted with Iranian families selected from a military center and the other with patients with chronic diseases and their relatives (Rocchi et al., 2017; Dadashi Haji et al., 2018).

Finally, the *Family Resilience (FaRE) Questionnaire* attempted to bridge all the gaps in the quantification of the family resilience construct in the oncological setting (Faccio et al., 2019). *FaRE* was validated for a population of patients with breast and prostate cancer and respective caregivers, in Italy, and presented acceptable psychometric properties. In the validation process, an initial 60-item version was refined into a 24 item questionnaire, which can be aggregated in four different factors: communication and cohesion, perceived social support, perceived family coping, and religiousness and spirituality (Faccio et al., 2019). In Portugal, breast cancer is the most frequent cancer diagnosis and the most frequent cause of cancer mortality, with incidence rates gradually increasing throughout the past decades (Forjaz de Lacerda et al., 2018). To the best of our knowledge, in this clinical population data regarding family resilience is scarce, and family resilience instruments lack proper psychometric evaluation. In that sense, here we propose to adapt and validate a short form of the European Portuguese version of the *FaRe* (FaRE-SF-P) for a sample of patients with breast cancer and their relatives, to better characterize family resilience in this context and to compare its psychometric properties with the original FaRE scale. We hypothesize that a Portuguese translation of the FaRE-SF will conserve the reliability and construct validity of the original version.

## Materials and methods

### Participants

Participants were recruited within the scope of the BOUNCE multicenter clinical study (Predicting Effective Adaptation to Breast Cancer to Help Women to BOUNCE Back) between April 2019 and January 2021 (Pettini et al., 2022). The study was conducted at the Champalimaud Clinical Centre and followed the same approach as the Portuguese Validation study of the Perceived Ability to Cope with Trauma (PACT; Lemos et al., 2022). Eligibility criteria included: female patients, 18–70 years of age at the time of diagnosis, histologically confirmed invasive early or locally advanced operable Breast Cancer (BC), tumor stages I – III, surgery included as part of the local treatment, receipt of any type of systemic treatment regardless of treatment type, and of adjuvant radiation therapy if indicated as part of local treatment. Criteria for exclusion were: presence of distant metastases, history of another malignancy or contralateral invasive BC within the last 5 years except cured basal cell carcinoma of skin or carcinoma *in situ* of uterine cervix, history of early onset (i.e., <40 years of age) mental disorder (i.e., schizophrenia, psychosis, bipolar disorder, major depression) or of severe neurologic disorder (i.e., neurodegenerative disorder), other serious concomitant diseases such as clinically significant (i.e., active) cardiac disease (e.g., congestive heart failure, symptomatic coronary artery disease or cardiac arrhythmia not well controlled with medication), myocardial infarction within the last 12 months, and/or major surgery for a severe disease or trauma which could affect patient’s psychosocial wellbeing (e.g., major heart or abdominal surgery) within 4 weeks prior to study entry, or lack of complete recovery from the effects of surgery.

For this validation study, conducted only with participants in Portugal, all participants were submitted to the same experimental research protocol, which included a baseline assessment for patients that started oncological systemic treatment approximately 3 months before. Longitudinal assessments were performed across 12 months, with two additional time points at 6 months (M6) and 12 months (M12). Since this study was performed with data collected in the BOUNCE Project, sample size calculation was dependent on the global aims of the main study, rather than performed to address the objectives of this sub-study. However, the minimum sample size recommended to perform this psychometric analysis, when considering the number of items and factors of the scale (Nunnally, 1978; Mundfrom et al., 2005), is less than the sample sized that was analyzed here.

### Measures

#### Sociodemographic and lifestyle questionnaire and medical data

This form includes questions on patients’ sociodemographic and lifestyle variables (age, educational level, marital status and employment status) and the characteristics of the disease and treatment (cancer staging and treatment type).

#### Family resilience questionnaire – short form

The FaRE-SF is a brief 12-item self-report questionnaire derived from two of the original FaRE subscales (Faccio et al., 2019): Perceived family coping (FaRE-PFC; 4 items −2, 5, 8 and 11), and Communication and cohesion (FaRE-CC; 8 items −1, 3, 4, 6, 7, 9, 10, 12). Answers are given in a Likert-type scale that ranges from 1 (“Totally disagree”) to 7 (“Totally agree”). The Perceived family coping scale refers to the ability to recover from a stressful life event by activating and mobilizing coping strategies to deal with the illness. Higher values means higher levels of perceived family coping (maximum value of 28). On the other hand, the Communication and cohesion scale measures the capacity of a family to be open to communicate about the illness, the associated feelings, their impact on daily life as well as their ability to think about ways to solve problems, conflicts and to share decision-making processes. Higher values in this scale means higher levels of family cohesion and communication, with a maximum value score of 56. While the FaRE-SF is not yet formally validated, in the original validation study of the full FaRE, good convergent validity values were found for both ‘Communication and Cohesion’ (rho = 0.56; *p* < 0.0001) and ‘Perceived Family Coping’ (rho = 0.30; p < 0.0001) scales when correlated with the Resilience Scale for Adults (RSA; Friborg et al., 2003).

#### Modified medical outcomes study social support survey – mMOS-SS

To assess convergent validity, we used the mMOS-SS (Moser et al., 2012) as a measure of Social Support. The mMOS-SS is a brief self-report Likert-type (1 = ‘never’ to 5 = ‘always’) scale with 8 items organized in two dimensions: emotional and instrumental social support (4 items each). This instrument presented very good psychometric properties, similar to those of the original 19-item from which it derived (Moser et al., 2012). The mMOS-SS validation study was conducted with three geriatric samples (two samples of women with breast cancer and one sample comprised by patients with chronic diseases) showing excellent reliability (0.88 < α < 0.93), a two-factor structure (instrumental and emotional social support) and a good convergent validity with the mMOSSS total scale (Hays et al., 1994). In this study we used the brief mMOS-SS scale based on the Portuguese version of the total scale by Alonso Fachado et al. (2007) to assess convergent validity, where Cronbach’s alpha values of 0.92 and 0.88 were obtained for the emotional support and instrumental support subscales, respectively.

#### Hospital anxiety and depression scale

The HADS is a 14-item measure of psychological distress divided in two scales with 7 items each: HADS-Depression, assessing symptoms of depression, and HADS-Anxiety, measuring symptoms of anxiety (Zigmond and Snaith, 1983). Higher score indicates higher levels of symptoms. In the oncological setting, the HADS is a widely used questionnaire, with several validation studies showing good psychometric properties (Mitchell et al., 2010). The Portuguese version was validated by Pais-Ribeiro et al. (2007) in a study including patients with cancer, with Cronbach’s alphas of 0.76 and 0.81 obtained for the Anxiety and the Depression subscales, respectively. In this study HADS was used to assess divergent validity.

#### European Organization for Research and Treatment of Cancer Quality of Life Questionnaire-Core 30

The EORTC QLQ-C30 is a measure of quality of life specifically developed and validated for the oncological setting. It includes 30 items divided in 15 dimensions: 5 functional scales (physical, role, cognitive, social and emotional functioning), 3 symptoms scales (fatigue, nausea and pain), a global quality of life scale and some single items to assess other symptoms, such as dyspnea, insomnia, appetite loss, constipation, diarrhea as well as the presence of financial difficulties due to oncological treatments. We used the Portuguese version of the EORTC QLQ-C30 in our study, validated by Pais-Ribeiro et al. (2008), in which the Cronbach’s alpha for the global quality of life scale was 0.88. Here we will focus more on the EORTC social functioning subscale that had a Cronbach’s alpha of 0.78 in the Portuguese validation study, to assess convergent validity.

### Procedures

Permissions to translate the FaRE-SF scale were obtained from the original authors by the BOUNCE consortium. We then followed the International Test Commission Guidelines for Translating and Adapting Tests [“ITC Guidelines for Translating and Adapting Tests (Second Edition),” Bartram et al., 2018]. The Portuguese version of the FaRE-SF was thus developed using a forward-backward translation process both from and to English and European Portuguese, as follows. (i) A forward translation was completed by two bilingual experts in Psychology of Portuguese dominant language, resulting in two translated versions of the FaRE-SF (FaRE-SF-1 and FaRE-SF-2). (ii) A translation panel composed of psychology and oncology specialists who had not been involved in any of the forward translations compared FaRE-SF-1 and FaRE-SF-2, and discrepancies were reconciled through discussion among the translators. (iii) The reconciled Portuguese translation of the FaRE-SF (FaRE-SF-3) was then back-translated into English by two bilingual official translators, of English dominant language, that were independent of each other, not involved in the original translations, and not familiar with the original scale, resulting in two independent back-translations (FaRE-SF-4 and FaRE-SF-5). (iv) The original translation team compared FaRE-4 and FaRE-5, resulting in a consensus back-translation version (FaRE-SF-6). (v) FaRE-SF-6 was then compared against the original FaRE-SF by the initial translation team, to identify any major differences between the two, resulting in a final review of the reconciled Portuguese translation (FaRE-SF-3), with adjustments leading to a synchronized version (FaRE-SF-7) (vi) In a cognitive debriefing session, FaRE-SF-7 was tested among a small group of patients, intended to represent the target population and language group (Portuguese Patients with Breast Cancer, *n* = 6) to assess if the respondents correctly understood the questions being asked, if the questions were clearly stated and if there were words or phrases that were not familiar. Minor suggestions were made by these patients essentially reflecting replacement of some words for synonyms with higher frequency in European Portuguese, so it could facilitate understanding. For example, “*pensamos*” (Portuguese word for “think”) was replaced by “*refletimos*” (Portuguese word for reflect), as the verb “to reflect” includes a sense of serious thought or consideration. Considering the input from these patients, the translation was reviewed and proof-reading was conducted to ensure that minor errors were corrected, resulting in the definition of the FaRE-SF final Portuguese version (FaRE-SF-P).

Study procedures and protocol were reviewed and approved by the Ethics Committee of the Champalimaud Foundation. All participants provided written informed consent, and the study was conducted in accordance with the tenets of the Declaration of Helsinki.

### Data analysis

Statistical analyses were performed using JASP version 0.14.1 (built on the R-package lavaan). Descriptive statistics were used for sample characterization. To assess dimensionality, a Confirmatory Factor Analysis (CFA) was conducted to compare a proposed solution based on two subscales from the original scale (Factor 1 – FaRE Perceived Family Coping; Factor 2 – FaRE Communication and Cohesion). To evaluate the goodness of fit of the tested factorial structure, we considered the following indices: non-significant χ2, CFI (comparative fit index), TLI (Tucker–Lewis index), and RMSEA (root mean square error of approximation), according to the suggestion of Schermelleh-Engel et al. (2003). The cut-off criteria proposed by the same authors were considered as indicative of goodness of fit, as follows: CFI and TLI good fit ~0.97, acceptable fit >0.95; RMSEA: good fit ≤0.05, adequate fit 0.05–0.08. Item local adjustment was assessed through the factor loadings (λ), which reflect the strength of correlation between the latent variable and the observed variable. We considered factor loadings above 0.40 as good indicators of the quality of the items (Gana and Broc, 2018). Reliability was assessed by internal consistency using Cronbach’s alpha and McDonald’s omega, with coefficients above 0.70 indicating good reliability (Hair, 2010). Corrected item-total correlation was also used, with values above 0.30 considered to be good (Cristobal et al., 2007). Pearson’s correlation coefficients were calculated between FaRE-SF-P subscales and mMOS – Emotional Support, mMOS – Instrumental Support and EORTC – Social Functioning scores for convergent validity; and with HADS total score, HADS-Depression and HADS-Anxiety for divergent validity. Finally, comparisons of the FaRE subscales scores between groups of treatment across study endpoints of assessment were analyzed by fitting a mixed model, with Geisser–Greenhouse correction, as implemented in GraphPad Prism 8.0.1, due to the presence of missing data. This model uses a compound symmetry covariance matrix, and is fitted using Restricted Maximum Likelihood (REML). In the presence of missing values, this method gives the same *p* values and multiple comparisons tests as repeated measures ANOVA, so the results can be interpreted likewise. Results with *p* < 0.05 were considered statistically significant.

## Results

### Descriptive statistics

Among the 163 patients who accepted to participate in the study, 147 completed all the questionnaires (Table 1). The majority of the participants included were middle age women (41 to 50 years old), with an overall mean age of 51.3 (SD = 9.1). More than a half of the participants had a graduate degree (73.5%), full or part-time employment (76.2%), and was married (74.1%). Regarding the ongoing treatment, at the moment of the assessment, 55.1% of the patients were under chemotherapy (CT) and 44.9% were undergoing endocrine therapy (ET).

**Table 1:** Demographic and clinical characteristics of the sample.

Demographic and clinical characteristics (*n* = 147)	*n*	%
Age, mean (SD)	51.3	9.1
Age group		
≤40 y	16	10.9
41−50 y	59	40.1
51−60 y	47	32.0
>60 y	25	17.0
Missing data	2	1.2
Education		
Primary	4	2.7
Lower secondary	7	4.8
Higher secondary	25	17.0
Post-secondary non graduate	3	2.0
Graduate degree	108	73.5
Marital status		
Single/Engaged	17	11.6
Married	109	74.1
Divorced/widowed	21	14.3
Employment status		
Employed	112	76.2
Unemployed/housewife	19	12.9
Retired	16	10.9
Treatment		
Chemotherapy (CT)	79	53.7
Endocrine Therapy (ET)	68	46.3

Descriptive statistics of individual FaRE-SF-P items are presented in Table 2, including mean, standard deviation, kurtosis and skewness. The same table provides the percentage of endorsement, showing a tendency for higher agreement responses (7 – “totally agree,” and 6 – “moderately agree,” respectively).

**Table 2:** Individual FaRE item summaries and reliability parameters.

Item	Statistics			Percentage of endorsement								Reliability		
	M (SD)	Sk	Ku	1	2	3	4	5	6	7	Total	Item-total correlation	α if item deleted	ω if item deleted
1	6.15 (1.35)	−2.17	4.58	2.0	2.0	2.7	4.1	4.8	29.9	54.4	100	0.76	0.90	0.91
2	6.32 (1.05)	−2.13	5.19	0.0	2.0	0.7	4.1	6.8	29.3	57.1	100	0.77	0.85	0.91
3	6.25 (1.20)	−2.45	7.09	2.0	0.7	1.4	2.7	8.8	28.6	55.8	100	0.85	0.89	0.90
4	6.48 (1.04)	−3.14	12.02	1.4	0.7	0.0	3.4	3.4	23.1	68.0	100	0.80	0.90	0.91
5	6.45 (1.08)	−3.27	12.76	2.1	0.0	0.7	2.7	2.1	27.4	65.1	100	0.82	0.87	0.87
6	6.06 (1.40)	−2.16	4.54	2.7	2.7	2.0	2.0	8.2	33.3	49.0	100	0.77	0.90	0.91
7	6.60 (0.89)	−3.09	12.50	0.7	0.0	0.7	2.0	6.8	13.6	76.2	100	0.57	0.91	0.92
8	6.42 (0.88)	−2.48	10.03	0.7	0.0	0.0	3.4	5.4	33.3	57.1	100	0.80	0.86	0.90
9	6.46 (0.89)	−2.18	5.81	0.0	0.7	0.7	3.4	5.4	27.2	62.6	100	0.70	0.91	0.92
10	6.23 (1.19)	−1.88	3.59	0.7	1.4	0.7	9.5	5.4	23.8	58.5	100	0.68	0.91	0.92
11	6.50 (0.85)	−2.65	10.04	0.0	1.4	0.0	2.0	3.4	33.3	59.9	100	0.81	0.85	0.90
12	6.63 (0.72)	−2.05	3.68	0.0	0.0	0.0	2.7	6.1	17.0	74.2	100	0.73	0.91	0.91

For each item of the FaRE scale, the mean (M), standard deviation (SD), and the percentage of endorsement for each possible item score (range 1–7) is presented. Sk = Skewness; Ku = Kurtosis; α if item deleted = Cronbach’s α if item deleted; ω if item deleted = McDonald’s ω if item deleted.

### Dimensionality

**Figure 1:**
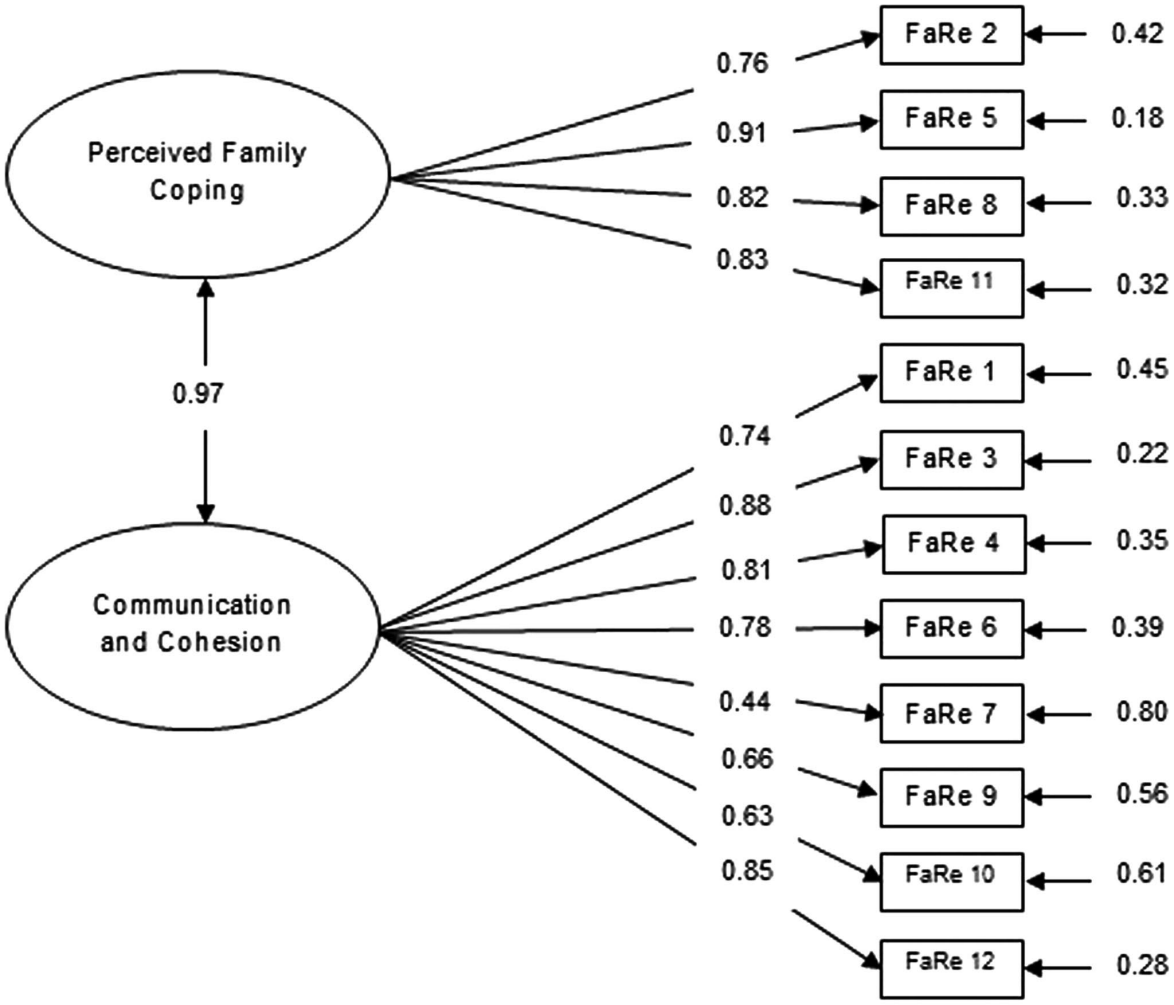
Confirmatory factor analysis of the FaRE two-factor model (Faccio et al., 2019) with standardized parameter estimates and measurement errors, in a sample of Portuguese women with breast cancer.

A CFA was performed to test the two-factor structure of the FaRE-SF-P consisting of Perceived Family Coping (Factor 1) and Communication and Cohesion (Factor 2) subscales. Goodness-of-fit indices of the general model have demonstrated good values and adequate fit for the study sample date, representing a two-factor structure: χ^2^ = 12.6; df = 53, *p* = 1.00; CFI = 1.00; TLI = 1.00; and RMSEA = 0.000. Furthermore, as presented in Figure 1, the two factors proved to be significantly positively correlated (*r* = 0.97, *p* < 0.001). Globally, in both factors, all items presented good local adjustment. In Factor 1 – FaRE-PFC loadings ranged from λ = 0.76 (item 2 – “*We believe that we can manage the illness*”) to λ = 0.91 (item 5 – “*We can work out the significant difficulties in our life such as this illness*”), while in Factor 2 – FaRE-CC loadings ranged from λ = 0.44 (item 7 – “*Everyone in the family feels free to express their own opinion regarding the illness*”) to λ = 0.88 (item 3 – “*In our family we feel that we can talk about how to communicate between us*”). In fact, only item 7 had a loading near to the recommended minimum value of 0.40. However, we decided not to remove it, as our tested model presented an overall good fit to the data, that did not improve with exclusion of item 7 (data not shown).

### Reliability

To assess internal consistency, we used the McDonald’s omega and the Cronbach’s alpha. Perceived Family Coping (Factor 1) showed an excellent reliability (ω = 0.92, 95% CI = 0.90–0.94; α = 0.89, 95% CI: 0.85–0.91). Corrected item-total correlations ranged from 0.77 to 0.87 and the internal consistency values decreased with removal of any item (Table 2). Communication and Cohesion (Factor 2) also presented excellent values of reliability (ω = 0.92, 95% CI = 0.90–0.94; α = 0.91 95% CI: 0.89–0.93). As depicted in Table 2, corrected item-total correlation coefficient values ranged between 0.68 and 0.85, with internal consistency remaining stable or decreasing with removal of any item.

### Construct validity

Analysis of the Pearson’s correlation coefficient (*r*) of the FaRE-PFC and the FaRE-CC with other constructs, to test convergent and divergent validity, are described in Table 3. Regarding convergent validity, both FaRE-PFC and FaRE-CC had weak but significant positive correlations with the MOS-Emotional Support (FaRE-PFC: *r* = 0.37, *p* < 0.001; FaRE-CC: *r* = 0.35, *p* < 0.001), the MOS-Instrumental Support (FaRE-PFC: *r* = 0.32, *p* < 0.001; FaRE-CC: *r* = 0.35, *p* < 0.001) and the Social Functioning scale from the EORTC QLQ-C30 (FaRE-PFC: *r* = 0.25, *p* < 0.01; FaRE-CC: *r* = 0.28, *p* < 0.001). For divergent validity, weak but significant negative correlations were found between FaRE subscales and the HADS-Total score (FaRE-PFC: *r* = −0.37, *p* < 0.001; FaRE-CC: *r* = −0.31, *p* < 0.001), HADS – Depression (FaRE-PFC: *r* = −0.37, *p* < 0.001; FaRE-CC: *r* = −0.31, *p* < 0.001), and HADS – Anxiety (FaRE-PFC: *r* = −0.29, *p* < 0.001; FaRE-CC: *r* = −0.26, *p* < 0.001).

**Table 3:** Correlations between FaRE-SF-P subscales and other psychometric measures to assess construct validity.

	FaRE – perceived family coping	FaRE – communication and cohesion
mMOS – EMOTIONAL support	0.37**	0.35**
mMOS – Instrumental support	0.32**	0.35**
EORTC – Social Functioning	0.25*	0.28**
HADS – Total Score	−0.37**	−0.31**
HADS – Depression	−0.37**	−0.31**
HADS – Anxiety	−0.29**	−0.26*

Pearson’s product moment correlation coefficient used as correlation measure. FaRE-SF-P = family resilience questionnaire – short form; mMOS = modified medical outcomes study social support survey; EORTC = European Organization for Research and Treatment of Cancer Core Quality of Life Questionnaire; HADS = Hospital Anxiety and Depression Scale.

** *p* < 0.001;

* *p* < 0.01.

### Perceived family coping and communication and cohesion in different groups of treatments across time

Mean scores of the FaRE – PFC and the FaRE – CC in our global sample are presented in Table 4.

**Table 4:** FaRE subscale scores at different time of assessments in the global sample and in both the chemotherapy and endocrine therapy treatment groups.

	Global sample			Chemotherapy sample			Endocrine therapy sample		
	*N*	Range	M (SD)	*N*	Range	M (SD)	*N*	Range	M (SD)
FaRE – PFC									
Baseline	146	9–28	25.8 (3.1)	78	13–28	25.8 (3.0)	68	9–28	25.7 (3.3)
6 Months from baseline	122	7–28	25.7 (3.2)	65	7–28	25.1 (3.7)	57	18–28	26.4 (2.4)
12 Months from baseline	116	7–28	25.5 (3.4)	62	15–28	25.2 (3.4)	54	7–28	25.8 (3.5)
FaRE – CC									
Baseline	147	15–56	50.9 (7.0)	79	15–56	50.4 (7.5)	79	21–56	51.4 (6.4)
6 Months from baseline	120	14–56	50.8 (6.4)	65	14–56	49.3 (7.7)	55	40–56	52.5 (3.8)
12 Months from baseline	115	20–56	50.3 (7.3)	62	23–56	49.3 (8.1)	53	20–56	51.4 (6.2)

FaRE – PFC = family resilience questionnaire, perceived family coping subscale; FaRE – CC = family resilience questionnaire, communication, and cohesion subscale.

**Figure 2:**
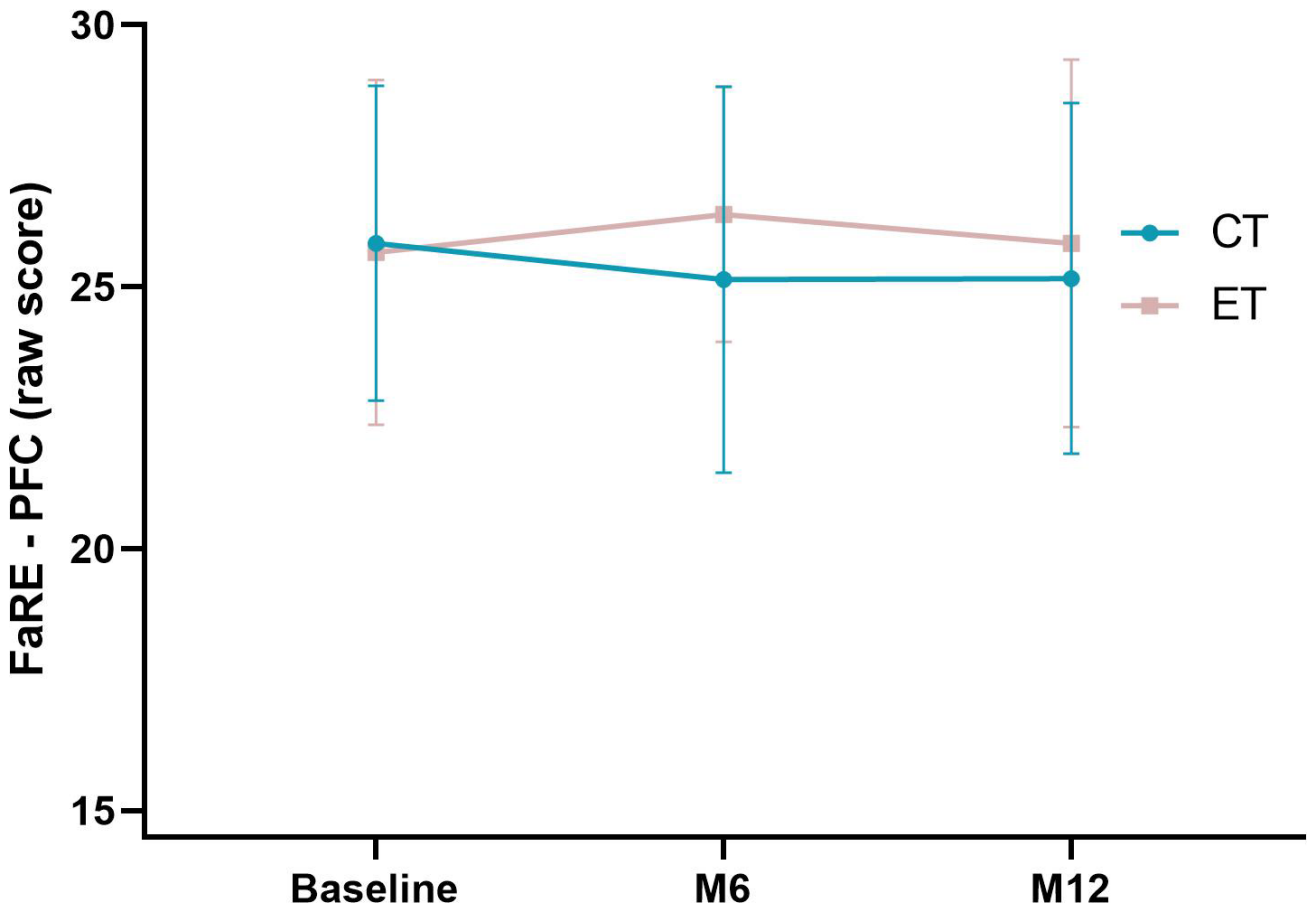
Mixed-models of the FaRE – PFC scores in both chemotherapy (CT; blue line) and endocrine therapy (ET; pink-gray line) in the three time points of the study (baseline, M6 and M12). Error bars represent the standard deviation.

**Figure 3:**
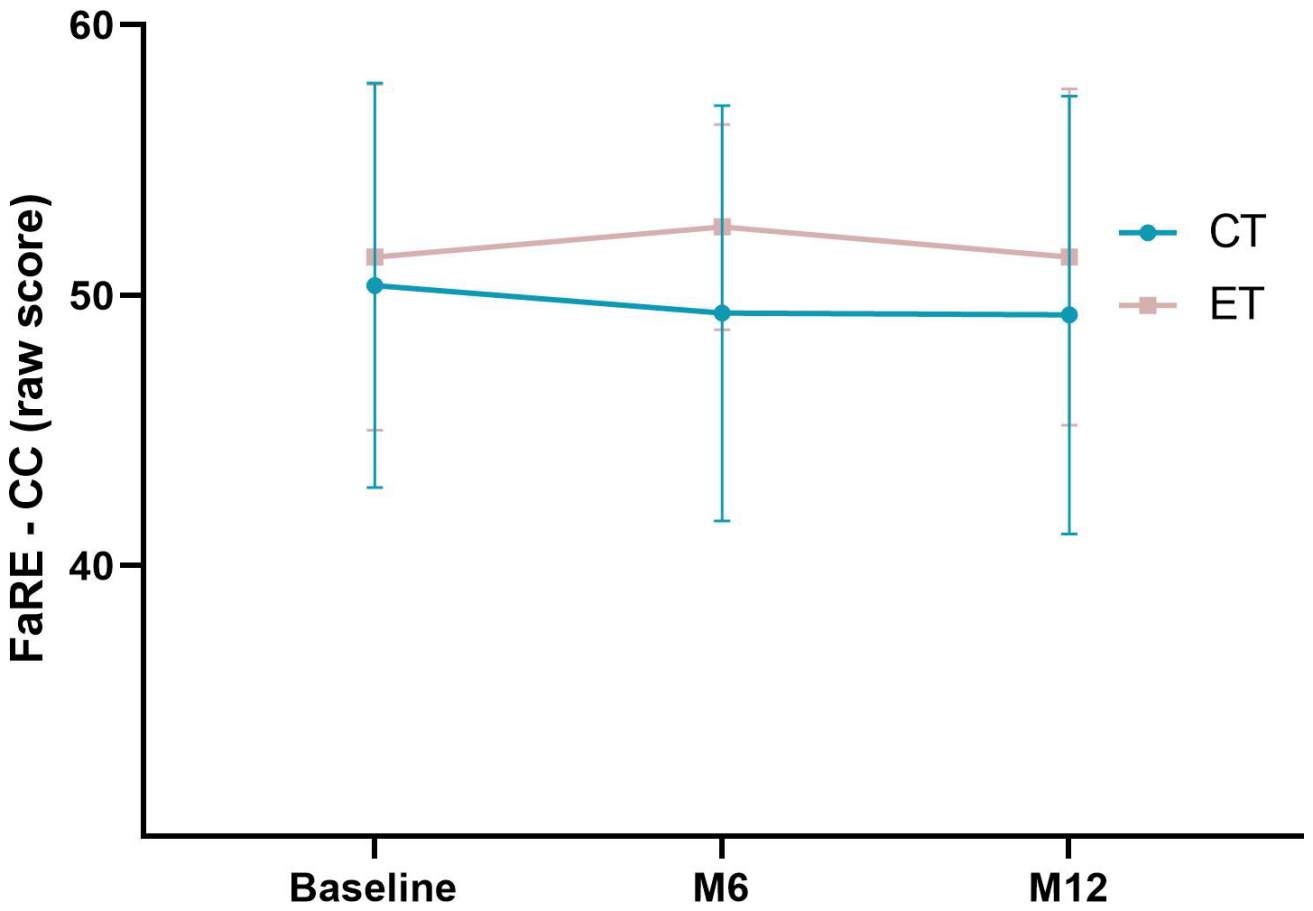
Mixed-models of the FaRE – CC scores in both chemotherapy (CT; blue line) and endocrine therapy (ET; pink-gray line) in the three time points of the study (baseline, M6 and M12). Error bars represent the standard deviation.

Mixed-models analysis were performed to assess the effect of type of treatment (CT or ET) and study time points (baseline, M6 and M12) on FaRE – PFC and FaRE – CC scores. Statistically significant effects were not found in FaRE – PFC for oncological treatment (*F*_(1,78)_ = 0.98, *p* = 0.33), time point of assessment (*F*_(2, 156)_ = 0.30, *p* = 0.74), nor the interaction between the two factors (*F*_(2,66)_ = 1.88, *p* = 0.16), suggesting that patient’s perceived family coping does not vary depending on the treatment nor on time (Figure 2). Similar results were found for the FaRE – CC scores: no significant differences depending on treatment (F_(1,78)_ = 3.05, *p* = 0.08), time point of assessment (*F*_(2,156)_ = 0.86, *p* = 0.43), nor the interaction between the two (*F*_(2,64)_ = 1.15, *p* = 0.32). These results suggest that family communication and cohesion seems to be relatively stable over time and similar between types of treatment, as illustrated in Figure 3.

## Discussion

The purpose of this study was to translate and culturally adapt and validate the Portuguese version of the FaRE-SF (FaRE-SF-P) for patients with early breast cancer. To the best of our knowledge, this is the first study validating the short version of the FaRE (Faccio et al., 2019). We demonstrate that the FaRE-SF-P is a valid measure to assess family resilience in this population, with good reliability and construct validity, and a two-factor structure reflecting Communication and Cohesion, and Perceived Family Coping subscales. Furthermore, we found that FaRE-SF-P subscales scores are stable over 1 year and did not differ between patients who underwent different types of systemic oncological treatment (CT and ET).

Our study assessed the psychometric properties of the FaRE-SF-P scale in a sample comprised by women with breast cancer, with an equivalent patient population also included in the validation study of the original FaRE scale (Faccio et al., 2019). Compared with the original full version of FaRE (Faccio et al., 2019), the FaRE-SF maintains the ‘perceived family coping’ and the ‘communication and cohesion’ subscales, excluding the ‘perceived social support’ and the ‘religiousness and spirituality’ subscales. The purpose of using a short form of the FaRE scale was to have a brief tool focusing mainly on the involvement of the family in problem solving and decision-making, as well as in the ability to rebound from a stressful life event, while keeping good psychometric properties. The excluded subscales focused more on family instrumental support and on the spiritual component of resilience, with the latter obtaining the lower percentage of variance on the FaRE total scale in the initial validation study (Faccio et al., 2019).

In our study, the CFA presented good evidence to support factorial validity, with a two-factor structure consisting of ‘Perceived Family Coping’ and ‘Communication and Cohesion’ providing excellent goodness-of-fit indices values, slightly better than the ones obtained in the FaRE original development and validation study with the four-factor structure (Faccio et al., 2019). In both subscales, no items with severe misfit were found. In terms of structural weights of the items, all had higher loadings than the recommended value of 0.40, with only item 7 from the FaRE – CC subscale (“*Everyone in the family feels free to express their own opinion regarding the illness*”) having a borderline loading value. Analyzing its content, item 7 is more related with the expression of personal opinions in a family context. The linguistic formulation of the remaining items seems to be more focused on a collective response or action (e.g., item 6 “*We think about the illness-related problems until we find a shared solution*”), which can explain this result. However, we decided to retain item 7 since it achieves the minimum recommended value for inclusion, the internal consistency of the scale did not improve with its removal and, furthermore, our model showed an overall very good fit. Regarding reliability, Cronbach’s α coefficient and McDonald’s ω were all above 0.89 in both FaRE – PFC and FaRE – CC subscales, which indicates high internal consistencies. Both subscales confirmed a good reliability which is aligned with the original FaRE version, where FaRE – PFC obtained a Cronbach’s α of 0.82 and FaRE – CC had a Cronbach’s α of 0.88 (Faccio et al., 2019).

Likewise, the construct validity of the FaRE-SF-P was supported in our sample of women with breast cancer. Indeed, both the FaRE-PFC and the FaRE-CC correlated significantly and positively with emotional and instrumental support (mMOS), as well as with social functioning (EORTC), thus supporting convergent validity. Moreover, they correlated significantly and negatively with distress, depression and anxiety measurements (HADS), therefore supporting divergent validity. Although these correlations were weak, they were statistically significant and had the expected directionality. There is no previous evidence of divergent validity of this scale. However, regarding convergent validity, the original FaRE authors tested the association between the total scale with another measure of resilience – the Resilience Scale for Adults (RSA; Bonfiglio et al., 2016) in their validation study (Faccio et al., 2019), confirming significant positive correlation (rho = 0.43, *p* < 0.0001). Specifically, the FaRE – PFC showed a significant positive weak correlation with RSA perceived family coping subscale (rho = 0.30, *p* < 0.0001), and the FaRE – CC demonstrated a significant positive moderate correlation with family cohesion from the RSA (rho = 0.56, *p* < 0.0001) (Faccio et al., 2019). As convergent validity of the FaRE subscales was already proved with a measure of resilience (RSA), in our study, we decided to use measures related to family resilience, but not necessarily measuring the same construct. As expected, patients with higher levels of perceived family coping, capable of communicating among them, and with a sense of family cohesion, have more emotional and instrumental social support and better social function. On the other hand, our findings are consistent with previous research that demonstrated that higher levels of resilience, even not specifically related to the family context, were associated with lower levels of depression and anxiety in patients with cancer (Min et al., 2013).

To summarize, in comparison with the original version of the scale (Faccio et al., 2019), the FaRE-SF-P conserved good psychometric properties, namely regarding reliability and the construct validity. Despite not having the same factor structure, which is expected since the FaRE-SF-P is a short version of the original scale, the two-factor structure comprising ‘Perceived Family Coping’ and ‘Communication and Cohesion’ subscales have slightly better goodness-of-fit indices values than the four-factor structure of the original FaRE scale (Faccio et al., 2019). Furthermore, these two subscales had good reliability both in the FaRE-SF-P and the original scale (Faccio et al., 2019), reflected, respectively, by a Cronbach’s α of 0.82 and 0.89 for the Perceived Family Coping subscale, and a Cronbach’s α of 0.88 and 0.91 for the Communication and Cohesion subscale. Convergent validity was assessed with different measures and both FaRE-SF-P and FaRE (Faccio et al., 2019) subscales had significant, yet weak, correlations with measures related to the construct of interest.

Furthermore, we found that perceived family coping and family communication and cohesion are stable over time and are similar between the two different systemic treatment groups (CT or ET). Family resilience was assessed in women with breast cancer starting approximately 3 months after initiating systemic treatment. As treatment phases can be characterized by distinct levels of uncertainty, differences across time in family resilience could be hypothesized. However, these results suggest that some of the family resilience processes that facilitate adaptation to cancer care (Faccio et al., 2018), such as the ability to activate coping strategies to deal with an illness within the family, or the family’s openness in communicating about this stressful event, are stable in time and do not differ between the different systemic treatment groups. In fact, the levels of resilience found shortly after diagnosis seem to remain stable across time, suggesting that family resilience may support the dynamic process in which patients and their families adapt coping strategies towards the ongoing challenges and uncertainty of cancer diagnosis and treatment. Therefore, the FaRE-SF-P proved to be a temporally reliable measure, applicable to patients with breast cancer independently of the type of systemic treatment or the phase of diagnosis and treatment. Importantly, as recommended by the original authors (Faccio et al., 2019), after testing the responsiveness of FaRE-SF-P to changes over 1 year, we can support its use as a psychoemotional tool in the course of cancer diagnosis and treatment.

Considering the aspects mentioned above, the FaRE-SF-P seems to have several benefits worth noting, that confirm its usefulness as a practical assessment tool of family resilience in the oncological setting. First, in the adaptation process, we followed a methodology based on international test commission guidelines [“ITC Guidelines for Translating and Adapting Tests (Second Edition),” Bartram et al., 2018], as well as the suggested quality criteria of health status questionnaires (Terwee et al., 2007), and of assessment tools in oncological settings (Tian et al., 2019). Second, the FaRE-SF-P has the advantage of being a short measure composed by 12 items with similar or better psychometric properties than other construct-related measures, such as the FRAS with 54 items (Sixbey, 2005), the FRA with 29 items (Duncan Lane et al., 2017), the WFRQ with 32 items (Walsh, 2016), and even the original FaRE (Faccio et al., 2019). The availability of a valid brief family resilience measure presents an opportunity to reduce patient burden in the oncological setting. Importantly, the FaRE-SF-P maintains the overall Walsh’s family resilience conceptual framework for functional adaptation of the whole family system to perceived adversity, with the exception of the spirituality component (Walsh, 2021). Previous evidence confirmed that family resilience has direct and indirect effects on quality of life and caregiver burden in patients with breast cancer (Li et al., 2019). Thus, the use of a reliable and valid measure of family resilience developed specifically for patients with cancer such as the FaRE-SF-P could help to develop prevention and intervention strategies, as has been highlighted by some authors (Hawley, 2000; Walsh, 2021). It is plausible that family resilience assessed by FaRE-SF-P might enable the prediction of affective symptoms, which could guide early referral to psychological and psychiatric consultations. This is emphasized by previous research indicating that families who struggle to activate resilience processes in face of a diagnosis of cancer tend to have increased levels of distress and higher risk of developing psychosocial problems (Weihs and Reiss, 1996; Kazantzaki et al., 2018). On the other hand, if there are difficulties in shared communication, problem-solving, emotional expression and mobilization of coping strategies, clinicians could develop specific interventions, supporting the patient and their family to develop their own meaning of the illness and integrating it in the family narrative.

Nevertheless, this study is not free from limitations. One is that we based our comparisons with results obtained in the validation study of the FaRE total scale. Even though FaRE and FaRE-SF-P share two subscales, comparisons should be cautious as different scales were applied to different populations of two distinct countries (Italy and Portugal). However, since there are no validation studies of the FaRE-SF-P, we believe it is important to have an overview of the differences and similarities between the psychometric properties of these two scales. Second, our sample size could be considered insufficient to power performance of a CFA. Even though we achieved the minimum necessary sample size condition for variables-to-factor ratio (Mundfrom et al., 2005), multigroup comparisons between oncological treatment types was not possible. Moreover, it was not possible to do a sample size calculation before the data collection once this study was performed under another multicenter study with a different aim. On the other hand, as FaRE-SF-P measures family resilience, it could be interesting to include a group of family members of patients with cancer to compare not only the psychometric properties between samples but also to assess the level of agreement between members regarding their resilience level, in line with the work developed by the original authors of the FaRE (Faccio et al., 2019). Finally, here we validated the FaRE-SF-P in a very specific population comprised by patients with early breast cancer. Despite the lack of significant differences between patients with breast cancer and patients with prostate cancer in the FaRE total scale validation reported by Faccio et al. (2019), it could be important to assess FaRE-SF-P psychometric properties in samples of patients with other tumor types and stages. Future studies should address this question by further validating the short form of the FaRE to different countries and to other clinical samples so healthcare professionals can properly assess family resilience and integrate this information in their specific oncological clinical practice.

In conclusion, we have demonstrated that the Portuguese version of the FaRE-SF is a reliable and valid measure of family resilience in patients with breast cancer, with a two-factor structure reflecting perceived family coping and family communication and cohesion. This study offers significant implications for both researchers and clinicians. The availability of a culturally validated instrument of family resilience, with good psychometric properties, will allow a better understanding of the importance of this construct in patients with cancer, as well as its impact on symptom burden, which should be addressed by further research. On the other hand, due to its brevity, the FaRE-SF-P can easily be included in oncological clinical practice without being significantly time consuming neither for the patient nor for the clinician.

## Data availability statement

The datasets presented in this article are not readily available because they belong to the BOUNCE Project Consortium. Requests to access the datasets should be directed to AO-M, albino.maia@neuro.fchampalimaud.org.

## Ethics statement

Study procedures and protocol were reviewed and approved by the Ethics Committee of the Champalimaud Foundation. Patients provided written informed consent to participate in this study.

## Author contributions

SA, RL, BS, and AO-M conceived and designed the work. RL and DF were responsible for the translation process of the FaRE scale. DF, BC, and BS acquired the data, including assessment of eligibility criteria of patients participating in the study. SA, RL, and AO-M analyzed and interpreted data. SA and BS extracted clinical and demographic data with input from RL, and AO-M. JG, TM, and AO-M supervised and validated the work. SA, DR, and AO-M drafted the manuscript, which was critically revised by the remaining authors for important intellectual content. AO-M supervised the research and acts as corresponding author. All authors contributed to the article and approved the submitted version.

## Funding

RL is supported by the 2018 Scientific Employment Stimulus from Fundação para a Ciência e Tecnologia, Portugal (CEECIND/04157/2018). DF, BC, BS, and AO-M were supported by the BOUNCE project (grant agreement number 777167), and DS and AO-M are supported by the FAITH project (grant agreement number 875358), both funded by the European Union’s Horizon 2020 research and innovation programme. AO-M is supported by grants FCT-PTDC/MEC-PSQ/30302/2017-IC&DT-LISBOA-01-0145-FEDER, and FCT-PTDC/MED-NEU/31331/2017, both funded by FCT/MCTES and the former co-funded by FEDER, under the Partnership Agreement Lisboa 2020 – Programa Operacional Regional de Lisboa. The funders had no role in the design of the study; in the collection, analyses, or interpretation of data; in the writing of the manuscript, or in the decision to publish the results.

## Conflict of interest

AO-M was national coordinator for Portugal of a non-interventional study (EDMS-ERI-143085581, 4.0) to characterize a Treatment-Resistant Depression Cohort in Europe, sponsored by Janssen-Cilag, Ltd. (2019–2020), and of trials of psilocybin therapy for treatment-resistant depression, sponsored by Compass Pathways, Ltd. (EudraCT number 2017-003288-36) and of esketamine for treatment-resistant depression, sponsored by Janssen-Cilag, Ltd. (EudraCT NUMBER: 2019-002992-33). He is also recipient of a grant from Schuhfried GmBH for norming and validation of cognitive tests.

The remaining authors declare that the research was conducted in the absence of any commercial or financial relationships that could be construed as a potential conflict of interest.

## Publisher’s note

All claims expressed in this article are solely those of the authors and do not necessarily represent those of their affiliated organizations, or those of the publisher, the editors and the reviewers. Any product that may be evaluated in this article, or claim that may be made by its manufacturer, is not guaranteed or endorsed by the publisher.
